# Medical Notes and Reflections

**Published:** 1857-05

**Authors:** 


### BOOK NOTICES.

*Medical Notes and Reflections.* By Sir HENRY HOLLAND, Bart., M.D. F.R.S., &c., &c. First American from the Third London Edition. Blanchard and Lea, Publishers.

This work comes to us in fine style, being plainly printed on excellent paper and nicely bound. It is a good sized volume containing nearly five hundred pages, divided into thirty-one chapters. Each chapter comprises the consideration of a different subject, making the work one of varied and extensive limit, and no definite idea of its contents can be conveyed by any written notice such as our limit will admit. In the preface to the Third Edition, he says, 'The earlier part of the volume will be found to relate chiefly to certain pathological principles, through which I have sought to associate together various morbid conditions, not usually thus connected in our systems of nosology. This method of inquiry, which I believe to conduct to many valuable results, I have further carried into the consideration of some particular disorders (Gout, Influenza, Dyspepsia. &c.), which at once illustrate the method in question, and are themselves best illustrated by it. The chapters comprised in the latter half of the volume relate more especially to the treatment of disease; including under this term not solely the consideration of particular medicines or remedial means, but also the methods in practice which a physician may expediently follow, and the duties he is bound to fulfil.'

The subjects discussed in the first fifteen chapters are as follows:—I. Medical Evidence; II. On Hereditary Diseases; III. Methods of Inquiry as to Contagion; IV. On diseases commonly occurring but once in Life; V. On the Connection and Classification of certain Diseases; VI. On Disturbed balance of Circulation, and Metastasis of Disease; VII. On the Influence of Weather in relation to Disease; VIII. On Diet and Disorders of Digestion; IX. On Gout as a Constitutional Disorder; X. On Morbid Actions of Intermittent Kinds; XI. On the Medical Treatment of Old Age; XII. On the Epidemic Influenzas of late Years; XIII. On Prognosis as a part of Practice; XIV. On Pain as a Symptom of Disease; XV. On Points where a Patient may Judge for Himself. The consideration of each of these subjects is interesting and instructive. His reasoning is plain, and rests on a basis of close and careful observation, made without any pre-conceived opinion or theory to substantiate by false experience. Chap. XIII. 'On Prognosis as a part of Practice," he closes by saying: "With all admission of exceptions, however, the rule by which the medical man may best abide, is that of adherence to what he believes to be true; and I repeat my belief that the likelihood of evil or injury from this source is far less than common apprehension makes it. Suspicion of a painful truth often disturbs much more than the truth plainly stated; and glosses of speech generally end in impairing the confidence which it is so essential should be maintained. These considerations will not appear frivolous to those who rightly estimate the dignity of the medical profession. They are especially needful to the young practitioner, whose course in this stage is beset with difficulties, under which many give way—capable of being removed only by a principal of conduct, and by good faith and firmness in sustaining it.'

The last sixteen chapters comprise the following subjects:—XVI. On Methods of Prescription; XVII. On Internal Hemorrhages and Morbid Secretions as the Subjects of Medical Treatment; XVIII. On some supposed Diseases of the Spine; XIX. On Hypochondriasis; XX. On the Exercise of Respiration; XXI. On some Points in the Pathology of the Colon; XXII. On the Action of Purgative Medicines; XXIII. On Bleeding in Affections of the Brain; XXIV. On the Use of Emetics; XXV. On the Use of Diluents; XXVI. On Sudorific Medicines; XXVII. On the Use of Opiates; XXVIII. On Mercurial Medicines; XXIX. On the Use of Digitalis; XXX. On Antimonial Medicines; XXXI. On the Hypothesis of Animalcule Life as a Cause of Disease—On Cholera—Conclusion.

The consideration of these subjects relate more particularly to the general treatment of disease, and the suggestions are made in a modest, unassuming manner, carrying force and reason with them. He has taken special pains throughout to make a plain distinction between ascertained facts and mere theoretical presumption; and whenever any conclusion is drawn from the latter, he has been cautious not to either convict himself or lead others to any definite result; he has rather sought to 'lead the way' for subsequent inquiries, and leave all questions open for further investigation. The last chapter 'On Animalcule Life as a Cause of Disease,' is one of peculiar interest. In the preface, he says: 'I have placed it at the end of the volume, as being in some measure detached by its more hypothetical character from the other subjects of which I have treated.' At the commencement of the chapter, he gives us to distinctly understand, that 'he is only defending an hypothesis in which the proof is one of presumption only.' And further on, he says: 'I have met with no opinion as to the causes and mode of propagation of the Epidemic Cholera, which can be considered to afford even plausible explanation of the facts; and in putting to trial the capability of another hypothesis to solve the phenomena of the disease, we are but seeking, by comparison of difficulties, to attain the conclusion which is least liable to them.' The reasoning of this chapter is throughout, fair and plainly open for discussion. His object is seemingly rather to provoke attention, and call out further research, than to force any conclusion on the reader. His ideas throughout the whole work are mainly original, and founded on extensive experience. We bespeak for it a liberal patronage, such as its merits may duly claim. G. K. A.

